# Fibrin Sheath Catheter-Related Endovascular Right-Sided Heart Infection in Heart Failure With Reduced Ejection Fraction: A Case Report

**DOI:** 10.7759/cureus.40060

**Published:** 2023-06-06

**Authors:** Javier B Chambi-Torres, Larri Rudman, Virendrasinh Ravat, Ivan S Gomez, George Michel

**Affiliations:** 1 Internal Medicine, Larkin Community Hospital, South Miami, USA; 2 Cardiology, Larkin Community Hospital, South Miami, USA

**Keywords:** right-sided infective endocarditis, catheter-associated infection, end-stage liver disease, heart failure with reduced ejection fraction, fibrin sheath infection

## Abstract

Patients with end-stage renal disease (ESRD) receive dialysis through either hemodialysis (HD) or peritoneal dialysis (PD). HD has challenges associated with vascular access and catheter-associated complications. The development of a fibrin sheath is a common complication with tunneled catheters. However, infection of the fibrin sheath is not usually encountered. We discuss the case of a 60-year-old female with ESRD and heart failure with reduced ejection fraction (HFrEF) receiving HD via tunneled right internal jugular (RIJ) Permcath who was diagnosed with an infected fibrin sheath located in the cavoatrial junction via a transesophageal echocardiogram (TEE). Compared to a transthoracic echocardiogram (TTE), a transesophageal echocardiogram provides a much more accurate diagnosis of this rare condition. Treatment primarily involves administering antibiotics based on sensitivity cultures and closely monitoring for any potential complications.

## Introduction

Tunneled catheters are widely used in hemodialysis (HD) and may result in several complications including thrombosis, fibrin sheath formation, infections, and central vein stenosis [1]. HD catheter malfunction is usually attributable to a catheter lumen narrowing and/or fibrin sheath formation around the tip [2]. If a previously functioning catheter develops issues later on, it is known as a late catheter failure [3], which may occur secondary to the formation of a fibrin sheath [4], which is essentially a circumferential endothelium sleeve formed around the catheter surface and remains within the vessel lumen after the removal of the catheter [5]. Right-sided infective endocarditis (IE) represents about 5%-10% of all cases of IE, and infective fibrin sheath extending from the superior vena cava (SVC) to the right atrium (RA) has been described as a cause of right-sided IE [6]. We present a case of a 60-year-old female with heart failure with reduced ejection fraction (HFrEF) and end-stage renal disease (ESRD) on HD in whom a fibrin sheath was discovered in the cavoatrial junction extending into the right atrium during a transesophageal echocardiogram (TEE) in the setting of central line-associated bloodstream infection (CLABI).

## Case presentation

A 60-year-old female with a past medical history significant for HFrEF, alcoholic liver disease with active alcohol use disorder, and ESRD on HD via tunneled right internal jugular (RIJ) Permcath was brought to the emergency department because of bizarre behavior. The patient was on hemodialysis three times a week with the most recent HD session five days prior. On admission, her vital signs were normal, and her urine drug screen was significant for opiates. Admission laboratory results were noted in Table 1.

**Table 1:** Pertinent laboratory results at the time of admission indicative of normocytic anemia, thrombocytopenia, elevated liver enzymes, and deranged renal function WBC: white blood cell, Hgb: hemoglobin, BUN: blood urea nitrogen, Na: sodium, K: potassium, AST: aspartate aminotransferase, ALT: alanine transaminase

Test	Reference range	Result
WBC	3.3-8.7 × 10^3^/uL	9.59 × 10^3^/uL
Hgb	12-16 g/dL	8.6 g/dL
Platelets	50,000-450,000/mm^3^	53,000/mm^3^
Ammonia	-	94 umol/L
BUN	6-20 mg/dL	93 mg/dL
Creatinine	0.6-1.3 mg/dL	3.83 mg/dL
Na	135-145 mEq/L	142 mmol/L
K	3.7-5.2 mEq/L	4.5 mmol/L
AST	8-33 U/L	76 U/L
ALT	4-36 U/L	43 U/L
Total bilirubin	0.1-1.2 mg/dL	2.9 mg/dL
Alkaline phosphatase	20-130 U/L	351 U/L

Physical examination revealed a regular heart rate without murmurs and bilateral leg edema. She was subsequently admitted to the ICU because of metabolic encephalopathy suspected to be secondary to severe uremia, which was managed by HD via a right internal jugular (RIJ) catheter. The patient’s ICU course was complicated by severe septic shock requiring dual vasopressors with norepinephrine and vasopressin to maintain hemodynamic stability. She was also started on vancomycin and meropenem empirically. Initial urine cultures from the day of admission (11/09/22) were positive for extended-spectrum beta-lactamase (ESBL) *Escherichia coli* (*E. coli*) sensitive to meropenem due to ESBL. As shown in Table 1, subsequent blood cultures (11/21/22 and 11/23/22) speciated to *Pseudomonas putida* and *Streptococcus mutans*, and tunneled RIJ Permcath tip cultures (11/24/22) were positive for *Pseudomonas aeruginosa*. As shown in Table 1, the minimal inhibitory concentration (MIC) for *Pseudomonas putida* and *Pseudomonas aeruginosa* was 0.5 for ciprofloxacin, and in the case of *Streptococcus mutans*, it was 1 for vancomycin.

The patient was started on ciprofloxacin 400 mg IV daily for two weeks and vancomycin 500 mg IV after every HD for six weeks under the supervision of the infectious diseases team. Details of cultures including urine, blood, and catheter tip are shown in Table 2.

**Table 2:** Bacterial culture results and sensitivities from different sites including urine, blood, and catheter tip N/A: not applicable

Date collected	Site	Result	Sensitivity (only resistance specified, sensitive to the rest of the classes of drugs)
11/09/22	Urine culture	Positive for *Escherichia coli*	Sensitive to most antibiotic classes
11/19/22	Blood culture	2/2 blood cultures grow gram-negative bacilli (*Pseudomonas putida*) and gram-positive cocci (*Streptococcus mutans*)	*Pseudomonas putida*: resistant to aztreonam, *Streptococcus mutans*: resistant to amoxicillin, penicillin G, cefotaxime, ceftriaxone
11/24/22	Catheter tip culture	Scant growth of *Pseudomonas aeruginosa*	Resistant only to aztreonam
11/29/22	Blood culture	No growth in five days	N/A
12/05/22	Blood culture	No growth in five days	N/A

Additionally, general surgery assisted with RIJ Permcath decannulation and placement of the right common femoral trialysis catheter on 11/21/22. As part of the initial workup, the patient underwent a transthoracic echocardiogram (TTE) on 11/08/22, which revealed a left ventricular ejection fraction between 15% and 20% with akinetic anteroseptal wall probably related to coronary artery disease, moderate mitral regurgitation, and mild aortic stenosis. Unfortunately, early in the patient’s course, guideline-directed medical therapy (GDMT) for HFrEF was not initiated as the patient was too hemodynamically unstable requiring dual vasopressors for hemodynamic support. As the patient’s hospital course progressed and the patient was discharged from the ICU, the patient did require the use of midodrine to maintain blood pressure support. With the expertise of cardiology services, the patient received 12.5 mg of carvedilol twice a day for two days, but it was discontinued because she needed vasopressor support during dialysis.

**Figure 1:**
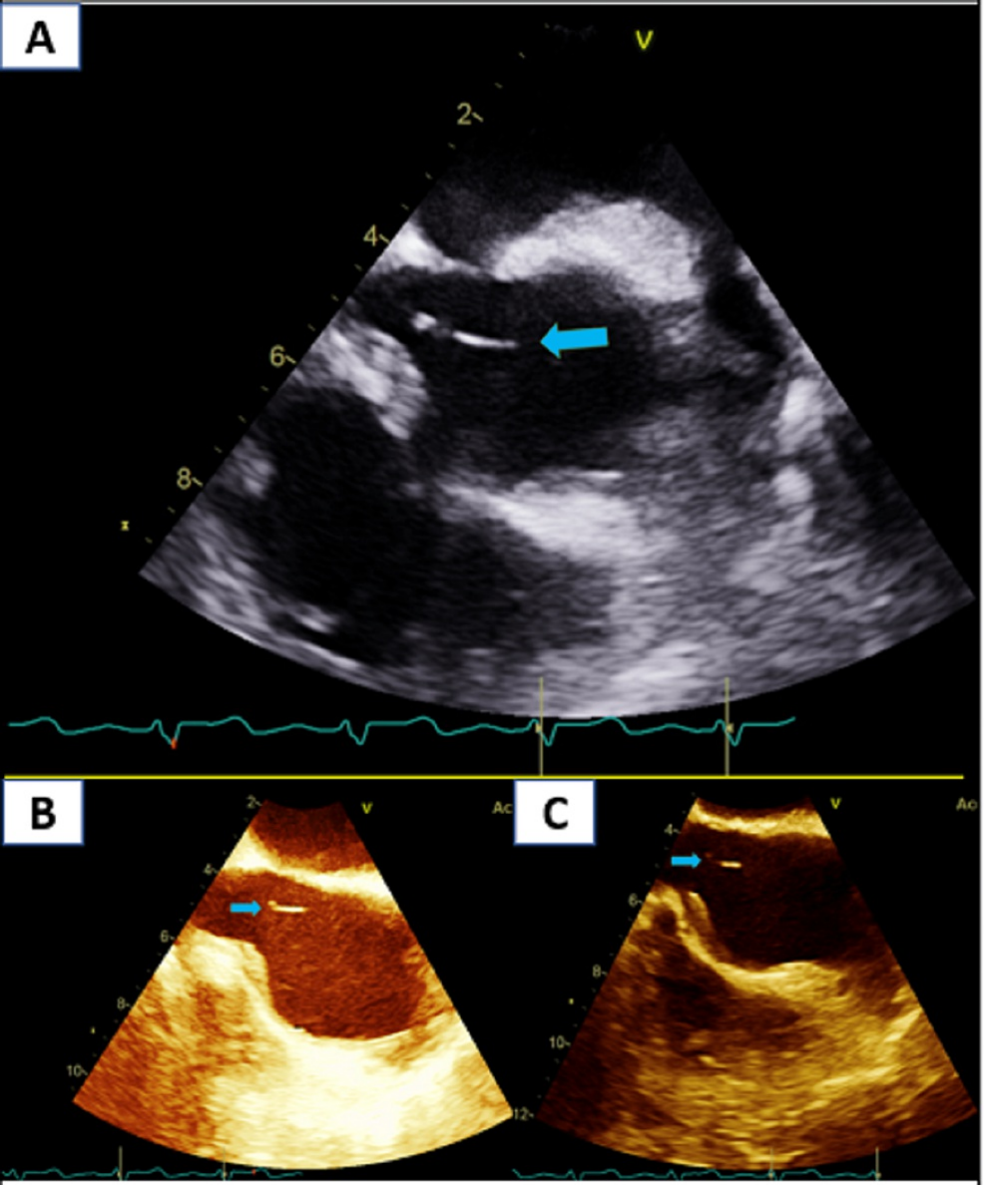
Fibrin sheath (blue arrow) in the cavoatrial junction (A-C)

In the setting of bacteremia, a TEE (11/23/22) was obtained, revealing a string-like material attached to the right atrium (Figure 1) with an ejection fraction of 25%. This finding is most likely a fibrin sheath from the previous right internal jugular catheter, and it can be associated with a fibrin sheath-associated endovascular infection of the heart in the setting of polymicrobial bacteremia (*Pseudomonas putida *and *Streptococcus mutans*) and positive catheter tip culture (*Pseudomonas aeruginosa*). The patient did convalesce from her illness and, at the time of discharge, was back at her physiologic baseline, New York Heart Association (NYHA) II, and was discharged home without oxygen with an indication for follow-up by cardiology service for the possibility of an automatic implantable cardioverter defibrillator (AICD) following cardiac catheterization after clearance by the infectious diseases team.

## Discussion

More than half of deaths in patients on dialysis are caused by cardiovascular disease whose progression is increased by factors such as atherosclerosis and left ventricular function reduction as kidney function decreases and hemodialysis further accelerates these factors [7]. Tunneled dialysis catheters are used for vascular access for HD through the internal jugular or common femoral vein, with optimal placement in the central circulation. Specific to our patient, optimal placement was achieved in the cavoatrial junction. The dysfunction rate of catheters by fibrin sheath was reported to be between 13% and 57% [1]. After the first 24 hours of catheter placement, a thin circumferential film of proteinaceous substance appears and may extend the entire catheter’s length for the next 3-7 days [8]. Animal models have shown that the fibrin sheath completely covers the catheter in its entire extent and circumference without any interruption [9] and that an important part of the sheath is composed of collagen and smooth muscle cells with overlying endothelial cells [10]. In humans, localized areas of venous endothelial wall injury were described after short-term catheter placement, and vein wall thickening with bridges between the catheter and the vein wall was observed in the long term [11].

The incidence of endocarditis is high in patients receiving HD using central venous access [12]. Fibrin sleeves can persevere after the indwelling catheter removal and trigger the development of thrombus or vegetation with the risk of distal embolization [13]. Transthoracic echocardiogram is not able to identify the infective endovascular fibrin sheath vegetations, but it is possible with the TEE if the vena cava is correctly focused [5]. In our case, the fibrin sheath was identified in the SVC-RA junction by TEE a few days after the removal of the right internal jugular tunneled catheter because of positive blood culture for *Pseudomonas putida* and *Streptococcus mutans*. In addition, the catheter tip culture was positive for *Pseudomonas aeruginosa*. We found five reported cases [14] of fibrin sheath-associated endovascular infections in the right side of the heart. The five cases and ours reported the location of the fibrin sleeve in the junction between the SVC and RA. The bacteria that caused the bacteremia in those five cases were methicillin-sensitive *Staphylococcus aureus*, methicillin-resistant *Staphylococcus aureus* (two cases), *Enterococcus faecalis*, and *Staphylococcus epidermidis*, and in our case, we identified *Pseudomonas mutans* and *Streptococcus mutans* in the blood and *Pseudomonas aeruginosa* in the tip of the catheter. Eleven reported cases [5] were treated with long-term antibiotics, five of them received anticoagulants, and two were treated with mechanical thrombectomy. Our patient was treated with two weeks of ciprofloxacin and six weeks of vancomycin tailored by culture data.

A meta-analysis showed that beta-blockers improved all-cause mortality in patients with chronic kidney disease and chronic systolic heart failure [15]. Our patient received carvedilol, a poorly dialyzed medication [16], for managing the HFrEF based on evidence illustrating mortality benefit in patients on dialysis with chronic heart failure [17]. However, she only took this drug for two days because of the vasopressor support requirement during dialysis, which means that the therapeutic options for the management of HFrEF were reduced to the possibility of AICD implantation after cardiac catheterization in case she was medically cleared by infectious diseases service.

## Conclusions

Fibrin sheath infection from a removed tunneled catheter is a rare cause of right-sided infective endocarditis, the most common location in the heart being the junction between the superior vena cava and the right atrium. Compared to a transthoracic echocardiogram, a transesophageal echocardiogram provides a much more accurate diagnosis of this rare condition. Treatment primarily involves administering antibiotics based on sensitivity cultures and closely monitoring for any potential complications. It is worth noting that the infective fibrin sheath did not acutely exacerbate cardiac function in our patient. In light of the scarcity of existing studies, it is essential to emphasize the need for additional research to enhance our comprehension and improve the management of this condition.
